# Supplementary material for: Nonlinear Information Bottleneck
Source: Entropy (Basel). 2019 Nov 30;21(12):1181. doi: 10.3390/e21121181 (PMC7514526; doi:10.3390/e21121181)
Supplement: Supplementary file 1 [file entropy-21-01181-s001.pdf]

Supplementary Material for "Nonlinear Information Bottleneck"

Artemy Kolchinsky, Brendan D. Tracey, and David H. Wolpert

In the main text, we report the result of optimizing bounds on the "squared-IB Lagrangian" [1], as defined both in nonlinear IB,

$$\mathbb{E}_{Q_\theta(Y,M)}\big[\log P_\phi(Y|M)\big] - \beta\big[\hat{I}_\theta(X;M)\big]^2, \tag{1}$$

and by extending the variational IB (VIB) [2] objective,

$$\mathbb{E}_{Q_\theta(Y,M)}\big[\log P_\phi(Y|M)\big] - \beta\big[D_{\mathrm{KL}}(P_\theta(M|X)\|R(M))\big]^2. \tag{2}$$

We consider three different datasets (MNIST classification task, FashionMNIST classification task, and the California housing prices regression task). In this Supplementary Material, we show results for the same three datasets, but now while optimizing bounds on the regular IB Lagrangian, as defined both by nonlinear IB,

$$\mathbb{E}_{Q_\theta(Y,M)}\big[\log P_\phi(Y|M)\big] - \beta\big[\hat{I}_\theta(X;M)\big], \tag{3}$$

and VIB,

$$\mathbb{E}_{Q_\theta(Y,M)}\big[\log P_\phi(Y|M)\big] - \beta\big[D_{\mathrm{KL}}(P_\theta(M|X)\|R(M))\big]. \tag{4}$$

Except for the change in objective, all parameters are the same as those reported in the main text. See the caption of Figure 1 in the main text for details on how to interpret the following info-plane diagrams.

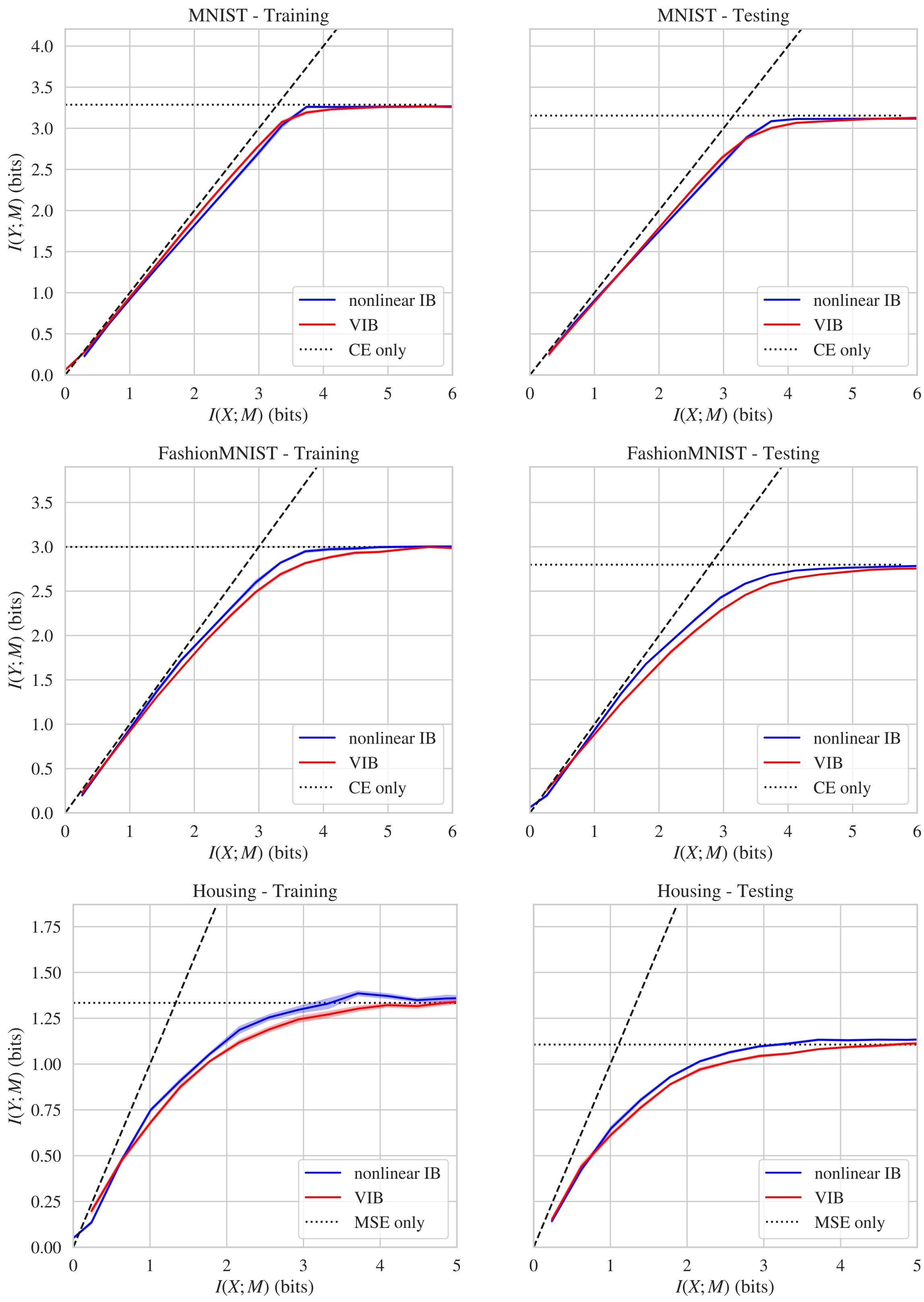

Figure S1: Performance of nonlinear IB and VIB when optimizing bounds on regular IB objective

[1] A. Kolchinsky, B. D. Tracey, and S. Van Kuyk, in *The International Conference on Learning Representations (ICLR)* (2018).

[2] A. A. Alemi, I. Fischer, J. V. Dillon, and K. Murphy, in *International Conference on Learning Representations (ICLR)* (2017).
